# Prognostic effect of pulmonary hypertension in patients with chronic kidney disease: Univariate and multivariate analyses of factors associated with survival

**DOI:** 10.3389/fmed.2022.972937

**Published:** 2022-10-05

**Authors:** Lei Wang, Wei Zhang, Cailian Zhang, Zhe Yan, Shaomei Li, Chunxia Zhang, Yakun Chen, Qing Pan, Xuzhi Liang, Xian Chen

**Affiliations:** ^1^Department of Respiratory and Critical Care Medicine, The Second Affiliated Hospital of Xi'an Jiaotong University, Xi'an, China; ^2^Emergency Department, First Affiliated Hospital of Xi'an Jiaotong University, Xi'an, China; ^3^Department of Pulmonary and Critical Care Medicine, Yan'an University Affiliated Hospital, Yan'an, China; ^4^Department of Nephrology, The Second Hospital of Hebei Medical University, Shijiazhuang, China

**Keywords:** pulmonary hypertension, chronic kidney disease, clinical characteristics, survival, prognostic effect

## Abstract

**Background:**

Prognostic effect of pulmonary hypertension (PH) in patients with chronic kidney disease (CKD) is not fully clear yet, this study was designed to elucidate baseline characteristics of CKD patients with different severities of PH, the association between kidney indicators and PH severity, and survival factors in CKD patients with PH.

**Methods:**

We extracted clinical data from electronic medical records of all patients diagnosed with PH in CKD from Jan 2016 to Dec 2020, and those with comorbid conditions causing PH were excluded. CKD stages were defined by estimated glomerular filtration rate thresholds. PH was defined as a systolic pulmonary artery pressure (sPAP) >35 mmHg estimated using echocardiograms. Demographics, clinical data, and test results were analyzed, and all-cause mortality data were obtained.

**Results:**

A total of 137 patients were included in the study. The mean age of the participants was 60 (42.5, 67) years, the mean sPAP was 58 (51, 69.5) mmHg, and 40.9% of the patients were women. Moderate PH group had more patients undergoing dialysis and higher frequency of coronary heart disease. Moderate-severe PH group had higher parathyroid hormone levels and lower low-density lipoprotein levels. Severe PH group had better kidney function parameters and lower serum phosphorus levels. PH severity had no direct relationship with CKD stages. In the univariate analysis, age and PH severity influenced survival. Multivariate analysis also showed independent prognostic effects for age and sPAP. Kaplan-Meyer curve intuitively displayed the survival differences among CKD patients with different PH severity. Predictor values of nomogram identified from survival analyses enabled calculation of death probabilities for CKD with PH patients. Nomogram was validated by ROC analysis.

**Conclusions:**

PH begins with early-stage CKD, and PH severity is not related to CKD progression. A higher pulmonary artery pressure and an older age are associated with an increased risk of death.

## Introduction

Pulmonary hypertension (PH) is a highly prevalent and important condition in adults with chronic kidney disease (CKD), and is often associated with poor outcomes in CKD patients, especially in end stage renal disease (ESRD) (1, 2), but it remains a neglected risk condition in renal patients. Previous studies noted that PH by echocardiographic criteria is prevalent among 21–27% of patients with CKD and up to 47% of patients with ESRD (3).

PH is classified into World Health Organization Group 1–5 (pulmonary arteriopathy, left heart disease, chronic pulmonary disease, chronic thromboembolic disease and unclear or multifactorial etiology, respectively) (4). PH in CKD is still an uncharacterized disorder, and the prevailing opinion is that PH in CKD patients falls under WHO Group 5 (5). The exact mechanisms of PH in this population remain poorly understood. Little is known about the pathophysiologic differences between PH in patients with CKD and others within the same PH groups. The possible pathogeneses of PH in CKD include an imbalance between vasoconstrictors and vasodilators, mineral-and-bone disorders, high-flow arteriovenous fistulas, anemia, recurrent pulmonary embolisms, fluid overload and left ventricular dysfunction (6–9).

The understanding of the characteristics and outcomes of PH in CKD patients is still incomplete, and published data on PH in patients with CKD are limited. Whether PH occurs only in ESRD patients is unknown, and how and in which stage PH originates are also unclear. Limited data are available for the earlier stages of CKD and the characteristics of CKD patients with different severities of PH, and early-stage CKD patients have been reported to have a functional abnormality of pulmonary circulation (10). Therefore, investigating the characteristics and outcomes of PH in CKD patients is crucial.

In this retrospective study, the characteristics and survival of patients with PH in CKD are described. We aim to (1) investigate the baseline characteristics of CKD patients with different severities of PH; (2) clarify the association between kidney indicators and PH severity; and (3) determine survival factors in CKD patients with PH.

## Materials and methods

### Study subjects

This unicentric retrospective study was performed in the Department of nephrology of Second Hospital of Hebei Medical University in China, where all patients received routine echocardiography examination during hospitalization. All patients diagnosed with PH in CKD were included from Jan 2016 to Dec 2020. The CKD etiologies included chronic glomerulonephritis, diabetic nephropathy (DN), hypertensive nephropathy, lupus nephritis, polycystic kidney disease, obstructive uropathy, renal amyloidosis, vasculitis with renal involvement and others (chronic interstitial nephritis, HELLP syndrome, light chain deposition disease and thrombotic microangiopathy) (Table 1). The inclusion criteria were as follows: (1) 18 years or older; (2) Diagnosis of CKD based on National Kidney Foundation-Kidney Disease Outcomes Quality Initiative (NKF-KDOQI) (11), including peritoneal dialysis and hemodialysis patients; (3) Diagnosis of PH when first hospitalized; (4) Complete clinical data. Patients were excluded according to the following criteria: (1) Connective tissue disease except for lupus, HIV infection, congenital heart disease, acute heart failure, portal hypertension and pulmonary veno-occlusive disease, and exposure to drugs and toxins; (2) Chronic obstructive pulmonary disease, interstitial lung disease and sleep apnea; and (3) Chronic pulmonary emboli.

**Table 1:** Etiology of CKD patients (*n* = 137).

**Variables**	**Number (%)**
Chronic glomerulonephritis	47 (34.3%)
Diabetic nephropathy (DN)	33 (24.1%)
Primary nephrotic syndrome	12 (8.8%)
Lupus nephritis	9 (6.6%)
Hypertension	8 (5.8%)
Vasculitis with renal involvement	6 (4.4%)
Polycystic renal disease	4 (2.9%)
Obstructive uropathy	2 (1.5%)
Systemic amyloidosis with renal involvement	2 (1.5%)
Henoch Schonlein Purpura	2 (1.5%)
Multiple myeloma	2 (1.5%)
Other	10 (7.3%)

The study received favorable opinion from the Ethics Committee of Second Hospital of Hebei Medical University and was performed in accordance with the Declaration of Helsinki. The study protocol and data collection instruments were submitted and approved by the Data Protection Commission of Hebei Medical University. All patients provided their written informed consent prior to inclusion in the study.

### Clinical and laboratory data collection

The patients' baseline characteristics (age, sex, smoking, area, disease duration), CKD etiologies, CKD stages (Table 2), dialysis status, comorbidities, and laboratory test results were recorded from our electronic medical record database.

**Table 2:** CKD stages.

**Stage**	**Description**	**GFR (ml/min/1.73 m^2^)**	**Action**
1	Kidney damage with normal or increased GFR	≥90	Diagnosis and treatment; treatment of comorbid conditions; slowing progression; CVD risk reduction
2	Kidney damage with mild decreased GFR	60–89	Estimating progression
3	Moderately decreased GFR	30–59	Evaluating and treating complications
4	Severely decreased GFR	15–29	Preparation for kidney replacement therapy
5	Kidney failure	≤ 15	Kidney replacement

### Echocardiographic evaluation

Doppler echocardiogram, as a routine examination, was performed for every admitted patient. Pulmonary hypertension was screened by echocardiogram to estimate pulmonary artery systolic pressure (sPAP), and for patients receiving dialysis treatment, Doppler echocardiogram was performed after dialysis. sPAP was estimated using a modified Bernoulli equation: sPAP =4 × (tricuspid systolic jet)^2^ + estimated right atrial pressure (RAP). When inferior vena cava (IVC) diameter ≤ 2.1 cm and collapses > 50% with inspiration, the RAP is assumed to be 3 mmHg (range, 0–5 mmHg). RAP is estimated to be 15 mmHg (range, 10–20 mmHg) when the IVC diameter > 2.1 cm with ≤ 50% collapse by inspiration, or 8 mmHg (range, 5–10 mmHg) for all other conditions (12). Pulmonary hypertension was defined as a sPAP >35 mmHg estimated using echocardiograms (13). Various sPAP cutoffs have been applied in previous studies, but a Doppler-derived sPAP of 35 mmHg is the most widely accepted threshold (14, 15). PH severity was categorized according to the sPAP as follows: mild (35–50 mmHg), moderate (50–70 mmHg) and severe (>70 mmHg) (16).

### Mortality data

The primary outcome of interest was all-cause mortality, which was ascertained from our electronic medical record database and telephone follow-up calls to patients or bereaved family members.

### Statistical analysis

We present continuous measurements as the mean ± SD if they were normally distributed or the median (IQR) if they were not, and categorical variables are presented as counts (%). The normality of variables was screened using the Shapiro–Wilk test. Means for continuous variables were compared using one-way ANOVA when the data were normally distributed; otherwise, the Kruskal-Wallis test was used. Proportions for categorical variables were compared using the χ2 test, and Fisher's exact test was used when the data were limited. Kendall's tau-b or Spearman's correlation analyses were used to examine relationships between renal function related variables and sPAP. The χ2 test and Mann-Whitney U test were used to compare various characteristics between the surviving and nonsurviving groups. Factors with statistical significance in univariate analysis or identified as having clinical significance by physicians were included in a multivariate analysis. Kaplan-Meier curves and log-rank tests were generated to illustrate the relationship between the PH severity and variables. Univariable and multivariable Cox regression analyses were performed to determine whether the prognostic of death was affected by other factors. Nomogram for predicting probabilities of death was built with R version 4.1.1. Receiver operating characteristics (ROC) curve analysis was used to calculate the area under the ROC curve (AUC), cut-off values, sensitivities, and specificities. All P-values were 2-tailed and were adjusted for multiple testing, and 95% CIs were reported. *P* < 0.05 was the threshold for statistical significance. All statistical analyses were conducted with SPSS version 22.

## Results

### Patient characteristics

**Figure 1:**
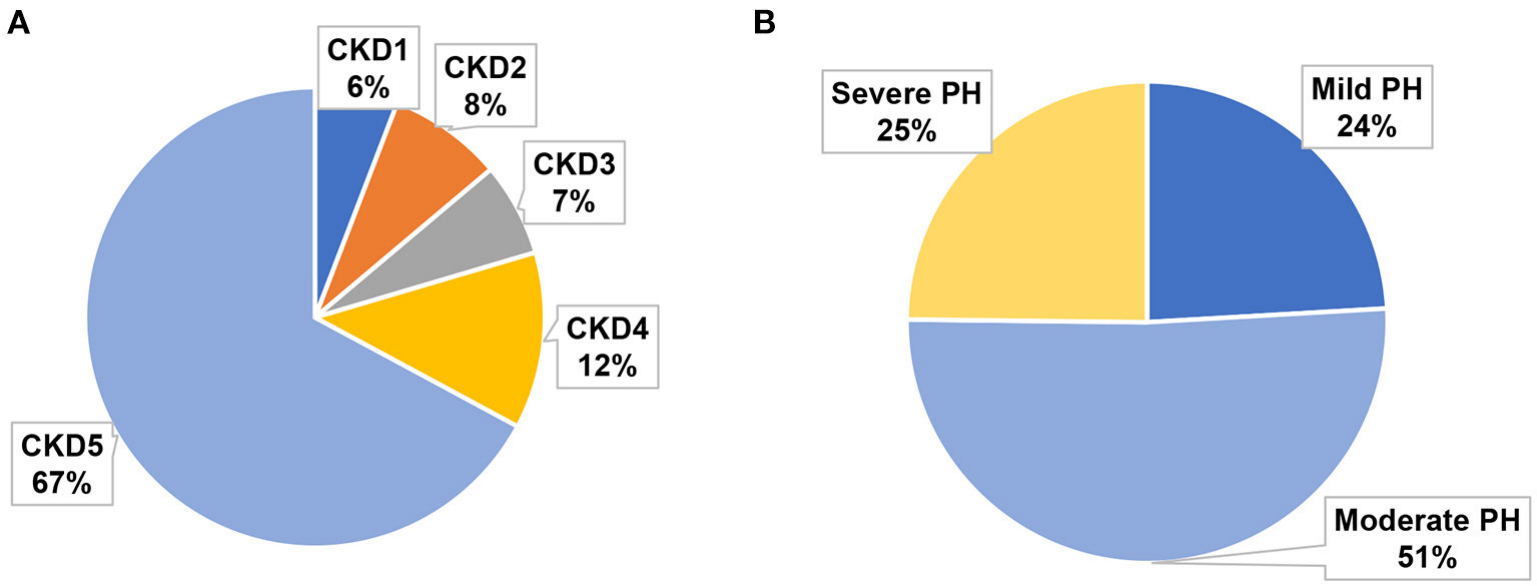
Proportion of different CKD stages and PH severities in the study cohort. **(A)** Proportions of CKD 1–5. **(B)** Proportions of mild, moderate, and severe PH.

**Figure 2:**
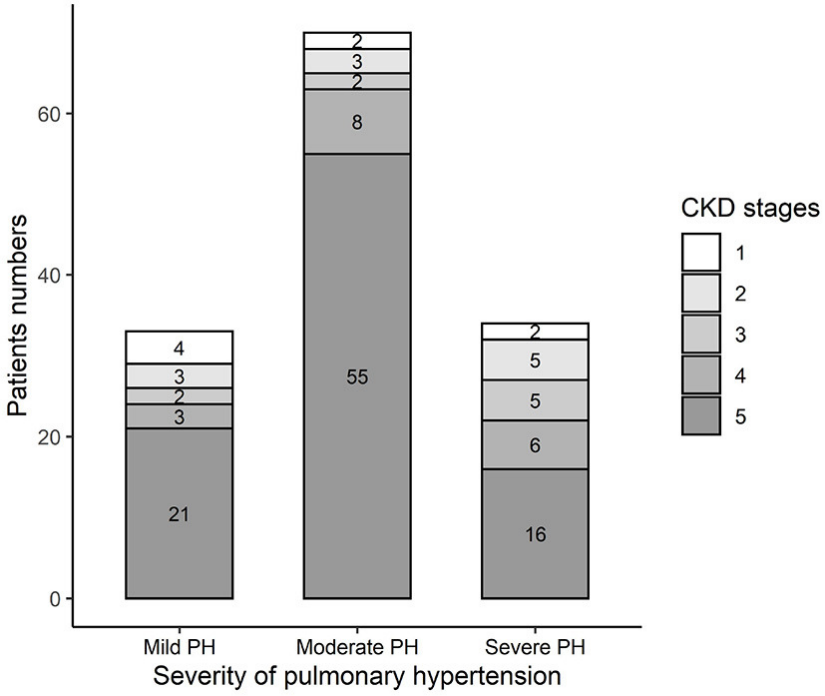
Distribution of different CKD stage in different PH severity groups in the study cohort. Mild vs. Moderate: *p* = 0.233; Mild vs. Severe: *p* = 0.391; Moderate vs. Severe: *p* = 0.014.

Table 3 summarizes the baseline characteristics of the study cohort, which consisted of 137 consecutive patients with PH in CKD. The primary diagnoses of the patients are shown in Table 1. The mean sPAP of the entire cohort was 58 (51, 69.5) mmHg. The mean age of the study cohort was 60 (42.5, 67) years, 40.9% of the patients were women, and 30.7% of the patients were from rural areas. Among 137 patients with PH in CKD, 8 (6%) patients had stage 1 CKD, 11 (8%) patients had stage 2 CKD, 9 (7%) patients had stage 3 CKD, 17 (12%) patients had stage 4 CKD, and 92 (67%) patients had stage 5 CKD (Figure 1A); mild, moderate and severe PH accounted for 24, 51, and 25% of the study population, respectively (Figure 1B). The distribution of PH severity and CKD stages are shown in Figure 2, no differences were found between mild vs. moderate PH and mild vs. severe PH, compared with severe PH, moderate PH group had more patients of CKD 5 (*p* = 0.014). Other clinical details are outlined in Table 3.

**Table 3:** Clinical characteristics of CKD with PH patients.

	**PH severity**			
	**Mild (*n =* 33)**	**Moderate (*n =* 70)**	**Severe (*n =* 34)**	***P***
**Gender**				0.822
Male	21 (63.6%)	40 (57.1%)	20 (58.8%)	
Female	12 (36.4%)	30 (42.9%)	14 (41.2%)	
**Age**	58.0 (39.0, 66.0)	59.5 (37.8, 69.0)	62.0 (54.5, 70.3)	0.365
**Smoke**				0.923
Yes	13 (39.4%)	25 (35.7%)	12 (35.3%)	
No	20 (60.6%)	45 (64.3%)	22 (64.7%)	
**sPAP** (mmHg)	44.0 (40.5, 46.0)	58.0 (54.0, 64.0)	75.0 (71.0, 80.5)	*** <0.001***
**Dialysis duration** (year)	1.8 (0.9, 2.7)	2.4 (0.5, 3.2)	1.2 (0.8, 3.0)	0.978
**Follow-up time** (year)	2.0 (0.9, 2.7)	1.8 (0.6, 3.2)	1.0 (0.7, 2.7)	0.397
**CKD duration**				0.154
<1year	16 (48.5%)	23 (32.9%)	9 (26.5%)	
1–2 years	5 (15.2%)	18 (25.7%)	9 (26.5%)	
>2 years	12 (36.4%)	29 (41.4%)	16 (47.1%)	
**Area**				0.389
City	7 (21.2%)	23 (32.9%)	12 (35.3%)	
Rural	26 (78.8%)	47 (67.1%)	22 (64.7%)	
**Renal disease**				0.712
Primary	16 (48.5)	28 (40.0)	15 (44.1)	
Secondary	17 (51.5)	42 (60.0)	19 (55.9)	
**Renal disease**				
non-DN	28 (84.8%)	49 (70.0%)	27 (79.4%)	0.222
DN	5 (15.2%)	21 (30.0%)	7 (20.6%)	
**Dialysis**				***0.004***
Hemodialysis	14 (42.4%)	44 (62.9%)	14 (41.2%)	
Peritoneal dialysis	0 (0.0%)	8 (11.4%)	2 (5.9%)	
No	19 (57.6%)	18 (25.7%)	18 (52.9%)	
**Arterio-venous fistula placement**				0.581
Yes	7 (21.2%)	20 (28.6%)	7 (20.6%)	
No	26 (78.8%)	50 (71.4%)	27 (79.4%)	
**24 h urinary protein** (24hUP, g/L)	3.0 (1.0, 5.8)	2.3 (0.9, 5.7)	1.7 (0.7, 5.9)	0.748
**Kidney function electrolyte**				
Cr (umol/L)	419.0 (160.0, 700.5)	571.0 (357.1, 809.4)	270.0 (113.3, 598.5)	***0.006***
BUN (mmol/L)	19.2 ± 12.1	23.9 ± 11.7	18.1 ± 11.6	***0.031***
eGFR (ml/min/1.73 m^2^)	11.8 (5.7, 37.0)	7.8 (5.4, 12.0)	17.5 (6.8, 44.9)	***0.010***
β2 microglobulin (mg/L)	10.8 (4.7, 17.4)	14.6 (10.1, 20.7)	11.4 (5.3, 19.8)	0.167
Uric acid (umol/L)	366.0 (254.5, 458.5)	385.5 (311.8, 512.3)	373.5 (307.3, 474.3)	0.487
Homocysteine (umol/L)	17.3 (11.7, 23.6)	22.2 (15.6, 28.2)	16.2 (11.9, 27.2)	0.177
PTH (pg/ml)	127.0 (28.6, 273.0)	235.0 (126.3, 428.5)	167.3 (73.1, 317.3)	***0.015***
Ca (mmol/L)	2.0 (1.9, 2.2)	2.1 (1.9, 2.2)	2.1 (1.9, 2.3)	0.493
P (mmol/L)	1.6 (1.2, 2.1)	1.7 (1.3, 2.2)	1.3 (1.1, 1.6)	***0.002***
**Blood lipids**				
CHOL (mmol/L)	4.4 (3.5, 5.6)	3.9 (3.3, 4.7)	3.7 (3.3, 5.1)	0.105
TG (mmol/L)	1.5 (1.2, 2.1)	1.3 (0.9, 1.8)	1.5 (0.9, 1.7)	0.173
LDL (mmol/L)	3.0 (2.0, 3.9)	2.3 (1.9, 2.9)	2.4 (1.8, 3.4)	***0.038***
HDL (mmol/L)	1.0 (0.9, 1.2)	1.0 (0.8, 1.2)	0.9 (0.7, 1.2)	0.612
**Blood routine**				
WBC (*10^∧9^/L)	6.2 (5.1, 7.9)	6.3 (5.3, 8.1)	6.7 (5.5, 8.1)	0.833
PLT (*10^∧9^/L)	173.0 (127.5, 230.5)	168.0 (123.8, 226.3)	139.5 (106.8, 188.3)	0.162
RBC (*10^∧12^/L)	3.0 (2.6, 3.7)	2.9 (2.5, 3.4)	3.4 (2.5, 4.0)	0.108
Hb (g/L)	94.0 (81.5, 107.5)	85.0 (74.8, 103.0)	103.5 (79.5, 120.8)	0.063
**Serum iron (umol/L)**	9.9 (7.4, 12.3)	7.6 (5.7, 11.2)	10.3 (5.9, 14.8)	0.163
**NT-proBNP (pg/ml)**	5,651.5 (1604.7, 16542.5)	14,400.0 (6,629.8, 20,800.0)	9,860.0 (2,010.0, 21,100.0)	0.194
**ESR (mm/h)**	34.0 ± 30.0	61.6 ± 36.1	56.5 ± 40.0	0.299
**PCT (ng/ml)**	0.2 (0.1, 0.6)	0.5 (0.2, 1.0)	0.3 (0.1, 0.8)	0.382
**Coagulation function**				
FIB (g/L)	3.8 (3.0, 4.2)	3.7 (3.0, 4.4)	3.6 (2.9, 4.2)	0.873
D-Dimer (ug/ml)	0.7 (0.3, 1.1)	0.6 (0.4, 1.0)	0.5 (0.2, 1.1)	0.327
FDP (mg/L)	4.4 (2.2, 10.5)	5.8 (3.2, 8.0)	4.1 (1.4, 10.0)	0.312
**Blood gas analysis**				
PH	7.3 ± 0.1	7.4 ± 0.1	7.4 ± 0.1	0.556
PO_2_ (mmHg)	80.3 (71.1, 88.9)	78.0 (67.5, 84.0)	66.9 (56.5, 81.8)	***0.025***
PCO_2_ (mmHg)	30.5 (26.0, 35.6)	28.7 (25.8, 36.6)	32.5 (28.3, 35.7)	0.379
**Comorbidities**				
**Hypertension**				0.407
No	9 (27.3%)	16 (22.9%)	12 (35.3%)	
Yes	24 (72.7%)	54 (77.1%)	22 (64.7%)	
**Diabetes**				0.342
No	24 (72.7%)	43 (61.4%)	19 (55.9%)	
Yes	9 (27.3%)	27 (38.6%)	15 (44.1%)	
**Tuberculosis**				0.427
No	33 (100.0%)	67 (95.7%)	34 (100.0%)	
Yes	0 (0.0%)	3 (4.3%)	0 (0.0%)	
**Tumor**				0.875
No	32 (97.0%)	67 (95.7%)	32 (94.1%)	
Yes	1 (3.0%)	3 (4.3%)	2 (5.9%)	
**Coronary heart disease**				***0.029***
No	28 (84.8%)	42 (60.0%)	20 (58.8%)	
Yes	5 (15.2%)	28 (40.0%)	14 (41.2%)	
**Bronchiectasis**				0.241
No	32 (97.0%)	70 (100%)	34 (100%)	
Yes	1 (3.0%)	0 (0.0%)	0 (0.0%)	
**Survive**				***0.010***
Yes	26 (78.8%)	47 (67.1%)	15 (44.1%)	
No	7 (21.2%)	23 (32.9%)	19 (55.9%)	

As showed in Table 3, in the study cohort, the moderate PH group included more patients undergoing dialysis (mild vs. moderate vs. severe: 42.4 vs. 74.3 vs. 47.1%, *p* = 0.004); the severe PH group had better kidney function than the mild and moderate PH group; the moderate PH group had the highest parathyroid hormone (PTH) level, followed by the severe PH group (mild vs. moderate vs. severe: 127.0 vs. 235.0 vs. 167.3 pg/ml, *p* = 0.015); the severe PH group had a lower serum phosphorus level (mild vs. moderate vs. severe: 1.6 vs. 1.7 vs. 1.3 mmol/L, *p* = 0.002); patients with moderate and severe PH had lower low-density lipoprotein (LDL) levels than the mild PH group patients (mild vs. moderate vs. severe: 3.0 vs. 2.3 vs. 2.4 mmol/L, *p* = 0.038); and hypoxemia worsened as the PH severity increased. The moderate PH group had higher frequency of coronary heart disease (mild vs. moderate vs. severe: 0 vs. 4.3 vs. 0%, *p* = 0.029); the mild PH group had highest survival rate (mild vs. moderate vs. severe: 78.8 vs. 67.1 vs. 44.1%, *p* = 0.01).

### Association between kidney indicators and SPAP

**Figure 3:**
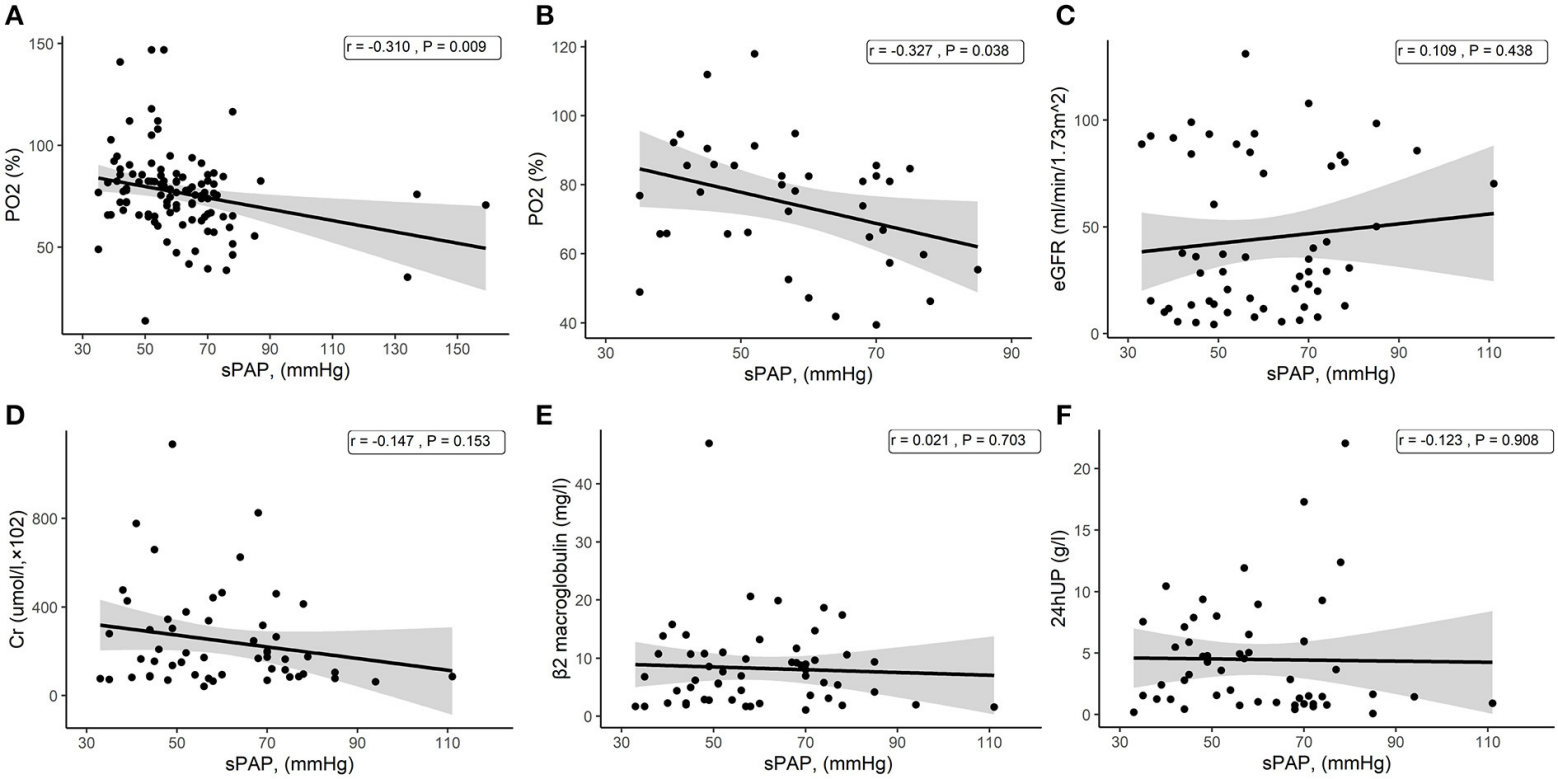
Scatter plots from the correlation analysis. **(A)** Correlation between sPAP and PO_2_ among all patients with PH in CKD. **(B)** Correlation between sPAP and PO_2_ among patients with PH in CKD without dialysis treatment. **(C)** Correlation between sPAP and eGFR among patients with PH in CKD without dialysis treatment. **(D)** Correlation between sPAP and Cr among patients with PH in CKD without dialysis treatment. **(E)** Correlation between sPAP and β2 macroglobulin among patients with PH in CKD without dialysis treatment. **(F)** Correlation between sPAP and 24hUP among patients with PH in CKD without dialysis treatment.

As mentioned above, the severe PH group had better kidney function, and we analyzed the associations between kidney indicators and sPAP in patients with PH in CKD without dialysis treatment. We did not find a significant correlation between sPAP and eGFR, Cr, β2 macroglobulin, or 24hUP (Figures 3C–F). Consistent with the above result, a negative correlation between sPAP and PO_2_ was found in all patients with PH in CKD (Figure 3A), including those without dialysis treatment (Figure 3B).

### Univariate and multivariate analyses of survival

Overall mortality was high, 35.8% of the patients died within an average of 23.29 ± 17.52 months follow-up. In the univariate analysis, the variables influencing survival were age and PH severity (Table 4). Advanced age was associated with death, but no significant associations were found with sex, eGFR, or disease duration and other index.

**Table 4:** Univariate analysis of factors associated with survival of all CKD with PH patients.

	**HR (95% CI)**	***P***
Age, year	1.05 (1.03, 1.07)	*** <0.001***
**Gender**		
Male	Reference	
Female	1.48 (0.85, 2.59)	0.170
eGFR (ml/min/1.73 m^2^)	1.00 (0.99, 1.01)	0.598
Hb (g/L)	1.01 (1.00, 1.01)	0.254
**NT-proBNP (pg/ml)**		
<17,000 (median)	Reference	
≥17,000	1.01 (0.57, 1.79)	0.973
**PH severity**		
Mild	Reference	
Moderate	1.53 (0.65, 3.56)	0.329
Severe	3.06 (1.28, 7.3)	***0.012***
**Dialysis**		
No	Reference	
Hemodialysis	1.02 (0.58, 1.81)	0.938
Peritoneal dialysis	0 (0,Inf)	0.997
**Kidney disease**		
Secondary	Reference	
Primary	0.56 (0.31, 1.02)	0.059
**Kidney disease**		
Non-DN	Reference	
DN	0.74 (0.36, 1.52)	0.414
**CKD duration**		
<1 year	Reference	
1–2 years	0.79 (0.36, 1.71)	0.550
>2 years	0.78 (0.42, 1.47)	0.446
**Coronary heart disease**		
No	Reference	
Yes	1.40 (0.79, 2.47)	0.249

The results of the multivariate analysis (Table 5) of all patients with PH in CKD showed independent prognostic effects for age and sPAP. For each 1 unit increment of age, an increased risk of mortality by 5 % (HR = 1.05, 95% CI:1.02, 1.07, *p* < 0.001), and for each 1 unit increment of sPAP, an increased risk of mortality by 3 % (HR = 1.03, 95% CI:1.01, 1.04, *p* < 0.001) were observed.

**Table 5:** Multivariable Cox regression analysis of risk factors associated with death in all CKD with PH patients.

	**Adjusted HR (95% CI)**	***P***
Age, year	1.05 (1.02, 1.07)	*** <0.001***
**Gender, vs**		
Male	Reference	
Female	1.77 (0.98, 3.20)	0.060
sPAP	1.03 (1.01, 1.04)	*** <0.001***
eGFR (ml/min/1.73 m^2^)	0.99 (0.97, 1.01)	0.317
Hb (g/L)	1.01 (1.00, 1.02)	0.128
**NT-proBNP (pg/ml)**		
<17,000 (median)	Reference	
≥17,000	1.20 (0.62, 2.33)	0.581
**Dialysis**		
No	Reference	
Peritoneal dialysis	0 (0, inf)	0.996
Hemodialysis	0.77 (0.37, 1.61)	0.486
**Kidney disease**		
Secondary	Reference	
Primary	0.69 (0.33, 1.44)	0.323
**Kidney disease**		
Non-DN	Reference	
DN	0.57 (0.24, 1.35)	0.201
**CKD duration**		
<1 year	Reference	
1–2 years	1.03 (0.44, 2.38)	0.946
>2 years	1.21 (0.56, 2.63)	0.630
**Coronary heart disease**		
No	Reference	
Yes	0.67 (0.33, 1.36)	0.269

**Figure 4:**
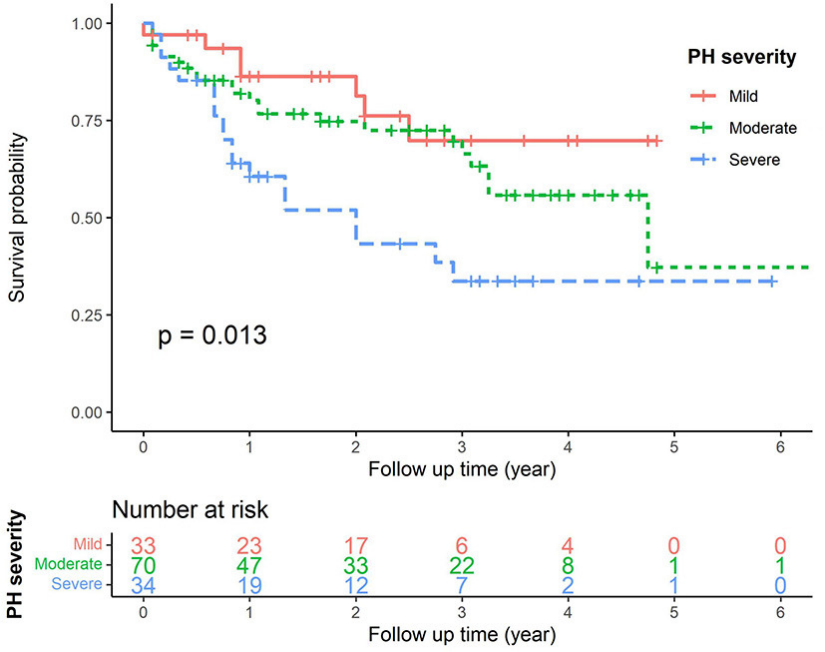
The Kaplan–Meier curves for CKD patients with different PH severity. *p* < 0.013.

In order to emphasize the prognostic effect of PH on CKD patients, we finally drew the Kaplan-Meyer survival curves (Figure 4) to show the effect of PH on patients' survival in this study. The different PH severity showed significant statistical differences (*p* = 0.013), indicating that PH as an important prognostic factor could affect CKD patients' survival.

### Predicted probabilities of death for all CKD with PH patients

**Figure 5:**
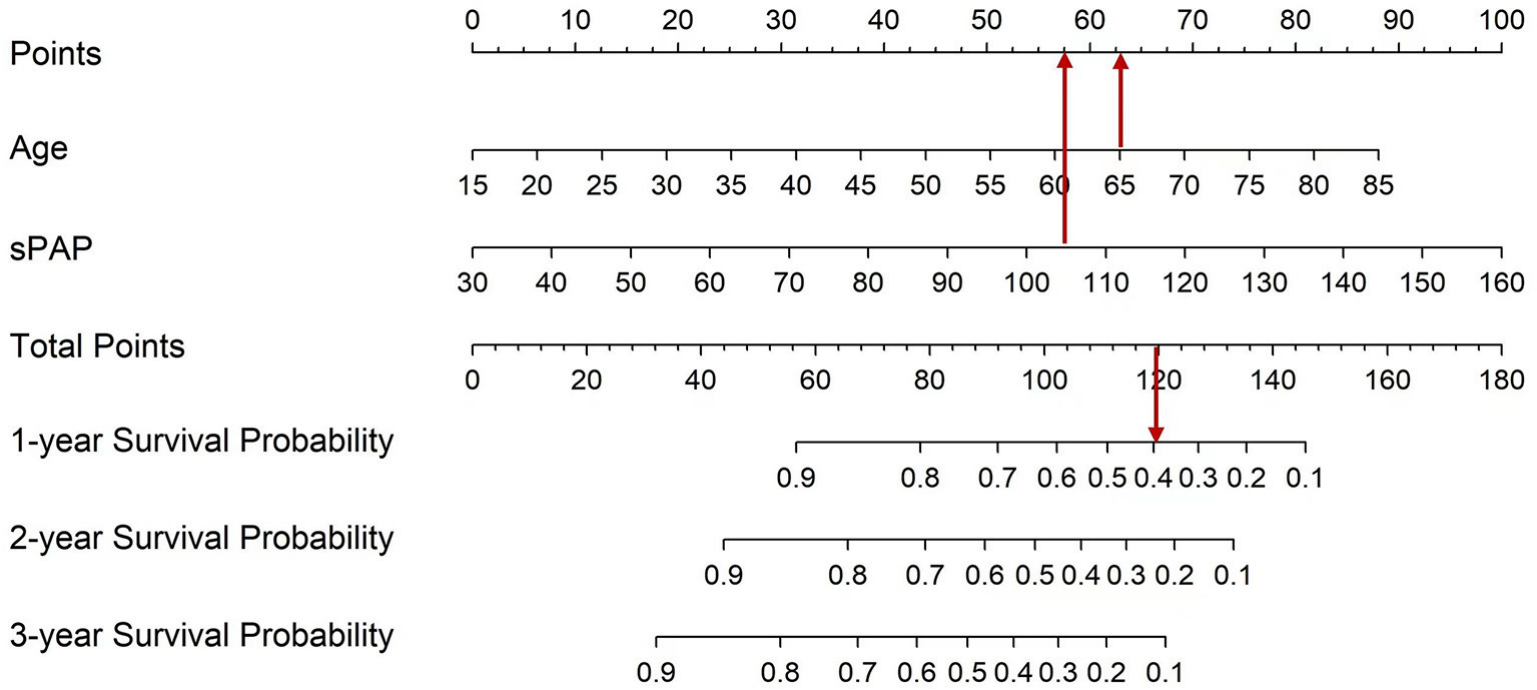
Nomogram which enabling calculation of predicted probabilities of death for all CKD with PH patients. To use the nomogram, a patient's values are located on each variable axis, and a line is drawn upward to determine the number of points received for each variable value. The sum of these numbers is located on the total point axis, and a line is drawn downward to the survival axes to determine the probability of survival at 1, 2, and 3 years.

To determine the survival probability of patients, nomogram associated with CKD-PH in this study was built (Figure 5). The points score associated with each predictor value were identified by reading up from that predictor value to the points scale at the top. Once a score has been assigned to each predictor value, a total points score is calculated. Translation from total points to the probability of the outcome is then made by reading down to the associated probability of the outcome from the total points scale. Therefore, using the nomogram, it can be seen that an individual aged 65 (62.5 points) with severe PH (105 mmHg, 57.5 points) has a total point score of 120. This equates to probability of 1-year survival of about 0.40. Determining the total score of the nomogram can enable us to more accurately and easily estimate the survival probability of patients, and timely intervene independent risk factors that affect the survival and prognosis of patients.

### Validation of the prognostic nomogram

**Figure 6:**
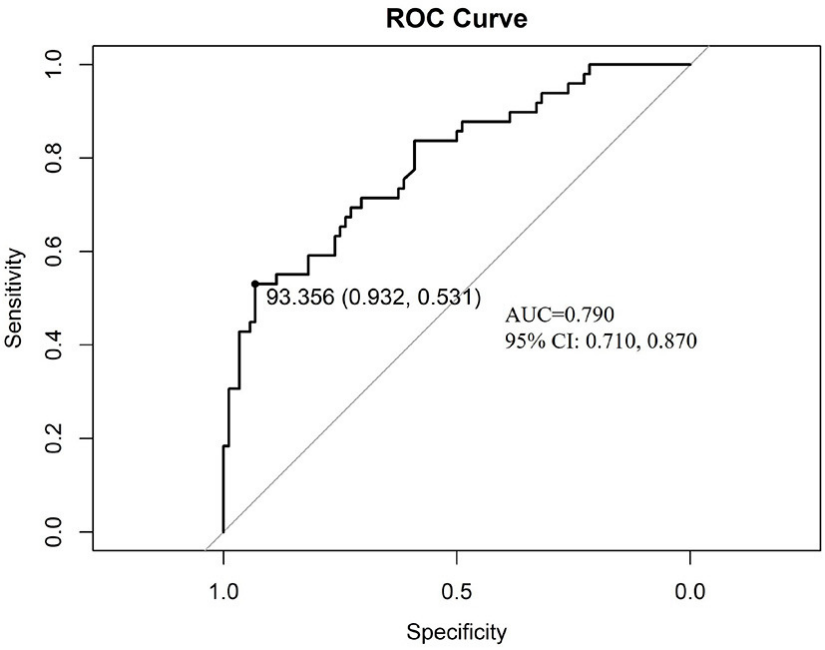
The discrimination test of nomogram model (ROC analysis). AUC is 0.790 (95%CI: 0.710, 0.870, *p* < 0.001), the cut-off value is 93.356 point with specificity 0.932 and sensitivity 0.531. ROC: the receiver operating characteristic; AUC: the area under the receiver operating characteristic curve.

ROC analysis was done to discriminate the ability of the nomogram, the area under the ROC is 0.790 (95%CI: 0.710, 0.870, *p* < 0.001), which proves the good performance of the nomogram. The best cut-off value is 93.356 point, the specificity is 0.932 and sensitivity is 0.531 (Figure 6).

## Discussion

The major findings of our study are as follows: (1) PH can be found in the early-stage of CKD, but most patients have moderate PH in CKD5; (2) increased iPTH levels, low serum phosphorus, low LDL levels, worsened hypoxemia and low survival rates were found in the advanced PH group; (3) no significant association was identified between kidney indicators and sPAP, and severe PH group had better kidney function parameters; (4) age and pulmonary artery pressure were associated with increased hazards for all-cause mortality.

Because previous epidemiologic data for PH in CKD patients are scarce and often with methodological limitations, such as retrospective data and/or small studies, the prevalence of PH among patients with CKD is difficult to estimate precisely. Previous studies mainly focused on the prevalence and mortality in ESRD (17), sPAP increases over time in patients with end-stage renal disease on maintenance dialysis, and the average increase in sPAP was 2.41 mmHg per year (8). Our results also showed that most PH patients were in the CKD5 stage. One study found that the PH prevalence was 23.76% among patients with a GFR ≥ 60 mL/min/1.73 m^2^, indicating that PH is not uncommon in patients with early-stage kidney disease (10). In this study cohort, PH also began in early-stage CKD, and PH severity was not related to CKD progression. These results indicated that unclear or multifactorial etiologies participated in the pathogenesis of PH in CKD, which requires further clarification. In routine clinical practice, physicians should be conscientious of PH in patients in different stages of CKD, and severe PH case can present in early stage. Data about PH severity distribution in CKD were few, one study showed that in all CKD patients, mild PH was most common, followed by moderate PH, and severe PH was the least, while the PH severity distribution was changed in different CKD stages (18). In this present cohort, patients with moderate PH were most, and CKD 5 was most common in different PH severity groups, especially in the moderate PH group.

Advanced CKD induces abnormal bone mineral metabolism where hyperparathyroidism and hyperphosphatemia promote vascular calcification (19, 20). Bone mineral disorder is a potential mechanism of PH pathogenesis in CKD, and one study showed positive correlations between echocardiographically estimated sPAP and calcium, phosphate and PTH in peritoneal dialysis patients (21). Our results showed that CKD patients with moderate-severe PH had higher PTH levels, but the presence of PH has also been reported to be unrelated to the PTH level or the severity of pulmonary artery calcifications in PH patients with chronic renal failure (19).

Hypocalcemia and hyperphosphatemia, are common in advanced CKD and are associated with an increased cardiovascular risk, increased mortality and CKD progression (22). No differences in serum calcium and serum phosphorus were found between CKD with PH and CKD without PH (23–25). In this study, lower serum phosphorus levels were found in the severe PH group with better kidney function parameters (Cr, BUN and eGFR), but whether these levels are related to the pathogenesis of PH in CKD is not known.

No significant association between kidney indicators and sPAP was found in this study cohort, and the severe PH in CKD group had better Cr, BUN and eGFR values, indicating that PH was not associated with kidney disease progression and that many other unclear or multifactorial etiologies were associated with PH onset. The prevalence of PH was high among dialysis patients (18). The process of hemodialysis itself may be a contributing factor for elevated PAP, but the exact cause is not known (23). In this study, the moderate PH group with more CKD5 patients (78.6%) included more patients undergoing dialysis (74.3%), but a high proportion of patients undergoing dialysis was not found in the severe PH group. To date, the effect of dialysis on the onset of PH is unknown, and future well-designed and large prospective studies are necessary to elucidate cause-effect relationship between dialysis and PH pathogenesis in CKD patients. A previous study demonstrated a significant association between malnutrition and PH in hemodialysis patients, and several lipid parameters, including total cholesterol and triglyceride, were decreased in patients with PH undergoing hemodialysis (24). The present study showed no differences in total cholesterol, triglyceride or HDL among mild, moderate and severe PH in CKD, while LDL levels were lower in moderate-severe PH in CKD. LDLs level have been reported to be decreased in PH patients, and human PH has been found to be associated with decreased LDL-receptor in the lungs (26). Low LDL may serve as an indicator of disease severity and prognosis in patients with PH, and LDL's anti-inflammatory properties may be relevant (27). We also found low LDL in CTD-PAH patients (28). Until recently, the exact mechanisms accounting for the association between low LDL and PH have been obscure, possible explanations include a chronic inflammatory state (29), malnutrition (30), and altered liver metabolism (31). Therefore, altered lipid metabolism in CKD patients may play an important role in PH pathogenesis, which requires further investigation.

PH in CKD is associated with cardiac disease, with increasing cardiac morbidity observed with increasing PH severity (32). Our results showed that coronary heart disease was more frequent in the moderate PH in CKD group, without increasing cardiac morbidity in the severe PH in CKD group. Prospective studies addressing patients with PH in CKD are needed to predict cardiac events.

The factors associated with a higher risk for death in PH in CKD1-5 (in the multivariate model) were age and sPAP in the present study. PH is a potential novel risk marker for CKD patients, and previous studies demonstrated that PH was associated with poor survival in CKD patients, especially ESRD patients (1, 3). Evaluation of pulmonary pressure might represent an important missing element for risk stratification of renal patients, and PH prevention appears to be an attractive target to improve survival in CKD patients.

PH had an independent prognostic effect, and recognizing and treating such patients as early as possible are vital to prevent life-threatening complications. In this retrospective study, the patients with PH had no records of any specific therapy for PH. Studies regarding “whether PH needs to be treated”, “what factors led to therapy”, “how to treat PH” or “how did treatment alter outcomes” in CKD patients are not available. Targeting PH might lead to improved outcomes in renal patients, but the lack of specific interventional studies and the need for more accurate evidence derived by adopting standardized methods to assess PH leave the issue open for future research. Limited evidence is available to recommend a specific treatment strategy for PH in CKD, and few studies of PAH therapies include patients with CKD or ESRD. Vasodilator therapy is the cornerstone of WHO Group 1 PH (PAH) treatment but has not been shown to have a benefit in WHO Group 5 PH, although studies investigating this issue are ongoing. Treatments targeting pulmonary vascular vasoconstriction and remodeling may be promising treatment options for select patients with CKD-associated pulmonary hypertension. Recent studies have shown that phosphodiesterase type 5 inhibitors have kidney protective, cardioprotective, and cerebrovascular protective roles, which can be used in kidney disease with systemic hypertension and acute and chronic kidney injury (33). ADDIN EN.CITE (34)Clinical trials are needed to determine if current and emerging treatments targeting endothelin-1, nitric oxide synthesis, prostacyclin, serotonin, and peroxisome proliferator-activated receptor g can improve outcomes for select patients with CKD-associated pulmonary hypertension.

This study had limitations. First, no measurements through right heart catheterizations (RHC) were performed to directly quantify PA pressure and PH due to left heart disease cannot be excluded completely, which is a limitation in this study and also in this field. Due to the invasive nature of RHC, most prior reports have used Doppler echocardiography to assess the presence of PH. In this study, PH was also diagnosed based on indirect echocardiographic estimates of PA systolic pressure. Although Doppler echocardiography is widely accepted for use as a screening tool in PH, the limitations of Doppler echocardiography are well known, and in some disease states, a higher discrepancy between echocardiographic and RHC data has been reported. Comprehensive studies including RHC should be conducted to characterize the prevalence of different types of PH in patients with early-stage CKD. Second, the etiology of pulmonary hypertension was not accurately determined. As a non-interventional trial, a cause-and-effect relationship between pulmonary hypertension and mortality cannot be established. Third, the sample size was relatively small, and more subjects had moderate PH than mild and severe PH. Thus, the data favor the moderate PH group due to the sample size. Fourth, cause-specific mortality and survival time were not analyzed, and a follow-up was not carried out to evaluate long-term survival. Last, because lack of awareness, not every CKD patient admitted screened for PH, the prevalence of PH in CKD patients in this study has not been estimated. This study showed that PH is an independent prognostic factor for CKD, routine screening for PH should be done among CKD patients in clinical practice.

## Conclusion

In summary, PH was common in CKD patients, PH is an independent prognostic factor for CKD, and a higher pulmonary artery pressure was associated with an increased risk of death. Future studies should define onset and development of PH during CKD, and explore the mechanisms underlying these associations to identify some potential therapeutic interventions for this high-risk population.

## Data availability statement

The raw data supporting the conclusions of this article will be made available by the authors, without undue reservation.

## Ethics statement

The study received favorable opinion from the Ethics Committee of Second Hospital of Hebei Medical University and was performed in accordance with the Declaration of Helsinki. The study protocol and data collection instruments were submitted and approved by the Data Protection Commission of Hebei Medical University. All patients provided their written informed consent prior to inclusion in the study.

## Author contributions

LW, XC, and CaZ: designed research. XC and WZ: analyzed data. XC, ZY, and QP: collected data. ChZ, YC, SL, and XL: participated in paper revision and data collection and check. LW: wrote the paper. All authors contributed to the article and approved the submitted version.

## Funding

This paper was supported by National Natural Science Foundation of China (81860014), the Fundamental Research Funds for the Central Universities (xzy012020060), S&T Program of Hebei (21377753D), Hebei Provincial Medical Science Project Research (20221078), and China Postdoctoral Science Fund (2021M702610). The funding body plays an important role in the analysis, and interpretation of data.

## Conflict of interest

The authors declare that the research was conducted in the absence of any commercial or financial relationships that could be construed as a potential conflict of interest.

## Publisher's note

All claims expressed in this article are solely those of the authors and do not necessarily represent those of their affiliated organizations, or those of the publisher, the editors and the reviewers. Any product that may be evaluated in this article, or claim that may be made by its manufacturer, is not guaranteed or endorsed by the publisher.
